# Industrial Transformation and Urban Economic Efficiency Evolution: An Empirical Study of the Yangtze River Economic Belt

**DOI:** 10.3390/ijerph19074154

**Published:** 2022-03-31

**Authors:** Yin Dong, Baishu Guo, Dawei He, Xiaoli Liao, Zhengyu Zhang, Xueqin Wu

**Affiliations:** 1 School of Public Administration, China University of Geosciences, Wuhan 430074, China; dongy_simlab@163.com (Y.D.); zhangzy_simlab@163.com (Z.Z.); 2 School of Arts and Communication, China University of Geosciences, Wuhan 430074, China; 3 Faculty of Resources and Environmental Science, Hubei University, Wuhan 430062, China; hedw09@163.com; 4 Hubei Geological Survey Institute, Wuhan 430030, China; liaoxl_dls@163.com; 5 Guishan Mountain Scenic Area, Wuhan 430050, China; hustwxq@163.com

**Keywords:** urban economic efficiency, industrial transformation, spatiotemporal variation, quantile regression, geographically weighted regression

## Abstract

Industrial transformation and high-quality urban development have become the core issues of urban-rural coordination and the leap forward in development in the new era. The research perspective of ‘pattern-process-mechanism’ is needed to reveal the spatiotemporal correlation characteristics of industrial transformation and urban economic efficiency evolution, and to expand its systematic, comprehensive and regional characteristics. Based on the geographical cognitive of local effects and spatial non-stationarity, we used a quantile regression model and a geographically weighted regression model to analyze the dynamic mechanism of industrial transformation and urban economic efficiency to explain the path characteristics of urban development and industrial transformation of the Yangtze River economic belt. The conclusions are as follows: (1) From 2000 to 2015, the average economic efficiency in the Yangtze River economic belt increased from 0.05 to 0.332, and the pattern gradually changed from spatial homogeneity to spatial mosaic; (2) From 2000 to 2015, the range and intensity of industrial transformation in the Yangtze River economic belt showed an increasing trend, while the speed of industrial transformation showed a downward trend, and the high-value unit of the three showed the characteristics of gradual homogenization; (3) From the perspective of the impact of industrial transformation on urban economic efficiency, the impact of the range and speed of industrial transformation on urban economic efficiency was gradually weakened, while the impact of the intensity of industrial transformation on urban economic efficiency was gradually strengthened, and the patterns of the three show the characteristics of a spatially inverted U-shaped distribution with high values in the middle reaches and low values in the upstream and downstream areas.

## 1. Introduction

The world is undergoing the largest wave of urbanization in human history, and the rural population is moving to cities at an unprecedented rate, causing a series of society and economic system problems including resource shortages, urban overloading, and environmental pollution which have had a huge impact on the sustainable development of cities [1,2]. In order to deal with the unsustainability of social development, some scholars call for the construction of a sustainable development analysis framework based on classical political economy, and then through multidisciplinary integration to put forward key countermeasures to achieve effective resource utilization, especially emphasizing the formation of utilization programs with efficiency as the core [3,4,5].

As the country with the fastest urbanization, China’s sustainable development faces the greatest difficulties and challenges. For this reason, General Secretary Xi proposed the concept of the new normal of the economy, which refers to sustainable economic development based on the symmetry of the economic structure [6]. The perspective of the "new normal" of the economy has determined that the scale and speed-oriented urbanization development model with ’small and medium-sized towns’ as the core is unsustainable, and it is necessary to turn to the quality-efficiency urbanization development model with ’megacities’ as the core [7,8,9] in order to realize the transformation of quality, efficiency, and refinement of cities under the limited supply of resources [10]. Therefore, the evaluation results of urban economic efficiency based on the input-output perspective of the urban system have not only become an intuitive reflection of the characteristics of urban development under the needs of the current era, but also an important basis for the overall coordination of urban space and the discrimination of regional functional advantages. At the same time, under the development demands of “quality change, efficiency change, and power change” as the core, it has gradually become a social consensus to guide industrial coordination and efficiency improvement, and to form a rational, advanced, and systematic non-agricultural economic industrial structure [11,12,13]. However, the current dynamic characterization of industrial transformation and industrial transformation at the macro level still lacks a systematic description [14,15,16]. In particular, the industrial transformation indicators in the dynamic analysis of urban economic efficiency are still relatively one-sided, mostly composite indicators such as the proportion of added value of secondary and tertiary industries, and do not consider the extent, speed, and intensity of industrial transformation to improve urban economic efficiency.

Urbanization and industrial transformation are the key components of disciplines such as geography, economics, and management. At present, the quantitative description of the effect of industrial transformation on the improvement of urban economic efficiency mostly adopts the perspectives and methods of economics and management, and lacks the summary and induction of the temporal and spatial perspectives of the driving effect from the perspective of geographical and spatial differentiation. At the same time, only incorporating a single dimension index into the research system of the urban economic efficiency improvement mechanism essentially ignores the sub-dimension characteristics of industrial transformation, and there is a risk of bias in the estimation of the direction and extent of the role of industrial transformation. The differences in thinking angles and cognitive methods have created barriers to knowledge interpretation and method application [17,18,19]. In particular, the research on the correlation between industrial transformation and urban economic efficiency based on the research perspective of “spatial pattern-time process-driving mechanism” in geography is slightly insufficient.

At present, most studies measure the regional economic output efficiency from the perspective of industrial classification, including the analysis of the dynamic mechanism of the urbanization process and the economic output efficiency based on the urbanization level measurement indicators of population urbanization, land urbanization and economic non-agriculturalization [20,21,22,23,24]. According to the economic non-agricultural characteristics of urbanization, some studies directly regard the economic output efficiency of decision-making units as urban economic efficiency, and carry out the analysis of the spatial and temporal pattern characteristics of urban economic efficiency at different scales and the analysis of the dynamic role of the factors that characterize the industrial structure. That is to say, it mainly follows the economic output efficiency improvement effect of evaluating the economic output efficiency characteristics of regional units based on industrial sub-dimensions and analyzing the industrial structure characteristics of urban units based on the dynamic mechanism framework [25,26,27]. Few studies focus on the analysis of the correlation characteristics between economic output efficiency and urbanization under the industrial classification, and most of them use the urban economic efficiency analysis framework based on economic and social benefit output capacity, aiming to provide scientific guidance for decision-making on “how to obtain maximum economic and social benefits with the minimum input of resource elements” [28,29,30].

## 2. Study Area

As the largest economic belt in China, the Yangtze River economic belt spans the east, the middle, and the west, and is formed by the Yangtze River passing through the adjacent provinces. It is located between 21°08′–35°20′ N, 97°21′–123°25′ E. As an economic zone that spans China’s three-level ladder, its altitude ranges from −491 to 7713 m, showing in its topographical features that the upper reaches are dominated by grasslands and mountains, the middle reaches are mainly mountains and plains, and the downstream areas are hills and plains. In addition, the climatic conditions in this area are very favorable, the average annual precipitation is 700–2000 mm, and the average annual temperature is 3–22 °C. In short, the area has excellent natural conditions for agricultural production and urban development.

**Figure 1:**
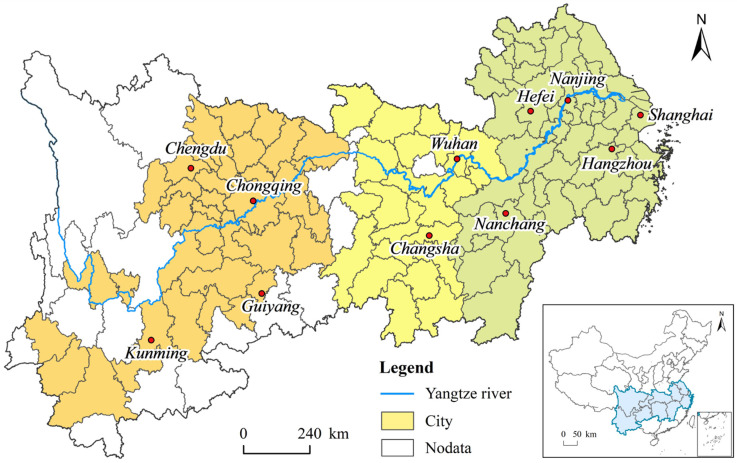
Geographical location and spatial composition of the Yangtze River economic belt.

At present, the Yangtze River economic belt has seen the formation of the Yangtze River Delta urban agglomeration with Shanghai as the core, the urban agglomeration of middle reaches of the Yangtze River with Wuhan as the core, and Chengdu-Chongqing urban agglomeration with Chengdu and Chongqing as the core (Figure 1), and its economy has accounted for more than 46% of China’s GDP. However, due to the strong historical evolution characteristics of the theme of the times, the Yangtze River economic belt has not been given timely development support and strategic leadership since it was proposed in the 1980s, resulting in the coexistence of backward economic development and stagnation for a long time. With the determination of the top-level strategy at the national level and the formation of a coordinated regional development mechanism, the ecological priority and green development model of the Yangtze River economic belt guided by “quality change, efficiency change, and power change” has become a path guide for its economic growth problem solving requirements, ecological environment harmonious coexistence appeal, and people’s pursuit of a better life value. It is worth noting that although the analysis of urban units in the entire Yangtze River economic belt is crucial for scientific decision-making, some autonomous prefectures have not released all of their statistical data, which has caused a serious problem of missing data. Therefore, this study mainly conducts an empirical analysis on 111 municipal units for which data are available.

## 3. Data and Methods

To explore the idea of quality development, we adhere to the context of time and space analysis, and based on the non-global and non-equilibrium geographical and spatial differentiation characteristics and dynamic driving mechanism system analysis, to describe the industrial transformation and urban economic efficiency evolution characteristics of the city unit of the Yangtze River economic belt. And based on the analysis, we can reveal the spatial correlation and mechanism of urban high-quality development and industrial transformation from the perspective of geographical time and space perspectives, the results of which have the value concept and policy connotation of high-quality development and rational promotion of industrial transformation. Based on this, first, because of the production boundary analysis framework, we use a three-stage data envelopment analysis (DEA) model that fully considers the heterogeneity of decision-making units and the difference of production frontiers’ natural breakpoint method that fully describe regional differences to portray the urban economic efficiency of 111 urban units in the Yangtze River economic belt in the fourth period from 2000 to 2015. Secondly, we measure the level of industrial transformation and the upgrading in the Yangtze River economic belt from the advanced industrial structure and the speed of transformation and upgrading, and use the natural breakpoint method to describe the spatial disequilibrium characteristics of industrial transformation at the municipal level. Finally, we use quantile regression and geographically weighted regression models to identify the mechanism of industrial transformation and upgrading on the spatial differentiation and stage differences of urban economic efficiency, with the hope that the research results and policy inferences will become a useful supplement to the analysis of urbanization and industrial transformation.

### 3.1. Variable Selection and Measurement

#### 3.1.1. Variable Connotation and Measurement

The calculation of urban economic efficiency includes the construction of the input-output index system and the selection of external non-equal environmental indicators. In the former, the output index is the non-agricultural output value that represents the output benefit of urban non-agricultural economic activities; the input indicators are the secondary and tertiary industry employees representing labor input, the fiscal expenditure and resource stock representing capital input, and the urban construction land representing urban land use. External environment indicators refer to existing research ideas, including the per capita disposable income of urban residents, which represents the income status of residents, the per capita GDP (gross domestic product), which represents the conditions of regional economic development, the total import and export of goods, which represents the regional economic exchanges with foreign countries, the total retail sales of social consumer goods, which represent the city’s overall spending power, and the number of students in ordinary colleges and universities, which represent the potential of social intellectual capital.

The driving mechanism analysis module includes the core explanatory variables of industrial transformation and control variables to avoid estimation bias. The former indicators include the industrial transformation range (x1), which is used to characterize the range of industrial transformation and upgrading, the Lilien index (x2), which characterizes the industrial transformation speed, and the Moore index (x3), which characterizes the industrial structure change intensity. Among the latter indicators, the number of students in ordinary colleges and universities (x4) is included as a representative indicator of the level of intellectual capital, so as to fully consider that intellectual capital is an important driving force and approach to stimulate production efficiency and industrial transformation; the proportion of fiscal expenditure (x5) in fiscal revenue serves as an indicator of the degree of government intervention, so as to fully reflect the potential impact of regional economic growth in the “GDP race” promotion game among government officials under the fiscal decentralization system [31,32]; the ratio of the per capita disposable income of urban residents to the net income of rural residents (x6) represents the income status of urban and rural areas, in order to take into account the spatial alienation and time polarization characteristics of economic growth and labor productivity caused by the concentration of labor and other social resources under the high urban-rural income gap.

#### 3.1.2. Data Resource

Considering the characteristics of development planning in stages and the cyclical changes in the economy and society, as well as the workload and complexity of data processing over a long period of time, we use the natural, social, and economic data of 111 cities in the Yangtze River economic belt from 2000 to 2015. Among them, the administrative boundary and geospatial data come from the resource and environment data cloud platform of the Chinese Academy of Sciences (http://www.resdc.cn/ (accessed on 20 August 2019)); the sources of humanistic and economic data include the China Urban Statistical Yearbook, the China Regional Economic Statistical Yearbook, the Provincial and Municipal Statistical Yearbooks, and the National Bureau of Statistics.

### 3.2. Models

#### 3.2.1. Three-Stage DEA Model

The three-stage DEA model based on the BCC (Banker, Charnes, and Cooper) model is widely used to measure the efficiency of urbanization, economic development, and agricultural production, which can effectively avoid the error of efficiency estimation caused by environmental factors in the analysis of the traditional DEA model. Therefore, the three-stage DEA model and natural breakpoint method were used to determine urban economic efficiency of the Yangtze River economic belt from 2000 to 2015, in order to fully analyze the spatial and temporal differentiation characteristics of urban economic efficiency. The BCC model assumes that there are *N* decision-making units that use the same input of type *I* to obtain the output of type *J* in time *T*, that is, the composition of input factors and that the output factors remain stable in different time periods. If it is mapped to the input-output index system, and x and y are used to represent the input and output indicators, respectively, the input and output data sets of decision-making unit *i* under the non-time dimension change can be written as *x_i_* = (*x*_1*n*_, *x*_2*n*_, *x*_3*n*_, …, *x_mn_*), *y_i_* = (*y*_1*n*_, *y*_2*n*_, *y*_3*n*_, …, *y_sn_*), the model under this data representation is expressed as follows [33]:

(1) $$(D_{\varepsilon }^{I})\left\{\begin{array}{l} \min[\theta -\varepsilon ({\hat{e}}^{T}s^{-}+e^{T}s^{+})]\\ \sum_{j=1}^{n}X_{j}\lambda_{j}+s^{-}=\theta X_{0}\\ \sum_{j=1}^{n}Y_{j}\lambda_{j}-s^{+}=Y_{0}\\ \lambda_{j}\geq 0,j=1,2,\ldots ,n;s^{-}\underline{\geq }0,s^{+}\underline{\geq }0\\ \sum_{j=1}^{n}\lambda_{j}=1 \end{array}\right.$$

In the formula, *θ* (0 < *θ* < 1) is the comprehensive technical efficiency in the VRS model; *λ_j_* is the weight variable; *s*^−^ (*s*^−^ ≥ 0) is a slack variable, which is used to represent the redundancy of effective production input; *s*^+^ (*s*^+^ ≥ 0) is the residual variable, which is used to represent the shortage of more efficient production output; *ε* is Archimedes infinitesimal.

Based on the basic assumptions of the stochastic frontier analysis (SFA) model, the input factor slack variables analyzed by the first-stage DEA model are re-evaluated, and they are divided into three types of factors: environmental factors, random factors, and technical factors, and then construct a composite function that can use statistical estimation parameters. The specific function model can be expressed as follows [34,35]:

(2) $$S_{ni}=f^{n}(Z_{i};\;\beta^{n})+V_{ni}+U_{ni}$$

In the formula, *n* represents the number of input elements in the traditional DEA model; *i* represents the number of decision-making units in the research object; *S_ni_* represents the difference between the actual input and the effective input of the *i*th decision-making unit, that is, the slack variable; *f_n_* (*Z_i_*; *β_n_*) is used to represent the influence of environmental factors on the slack variable. The Cobb-Douglas production function is often used in the SFA model, and other production functions can also be set according to theoretical assumptions, model testing and parameter analysis. Among them, *Z_i_* and *β_n_* are set to evaluate the environmental variables and their parameter vectors set in the system, respectively; *V_ni_* is used to reflect the effect of random factors on slack variables, and is often set as *V_ni_* ∈ N (0, *σ_vn_*^2^); *U_ni_* is the unilateral error term rate used to reflect management factors, which is often set as *U_ni_* ∈ N (*μ_u_*, *σ_un_*2), and the assumption that *V_ni_* and *U_ni_* are independent and irrelevant must be satisfied. Through the second-stage SFA-like model, the maximum likelihood estimation is used to measure the values of parameters such as *β_n_*, *σ*^2^, *γ*, *V_ni_* and *U_ni_*, and the input value under the homogeneous environment is obtained through the basic assumptions and model settings of the SFA-like model. The basic formula is expressed as follows:

(3) $$\begin{array}{c} X_{ni}^{*}=X_{ni}+[\max(Z_{i}\beta^{n})-Z_{i}\beta^{n}]+[\max(V_{ni})-V_{ni}]\\ n=1,\;2,\;\ldots ,\;N;\;i=n=1,\;2,\;\ldots ,\;I \end{array}$$

In the formula, $X_{ni}^{*}$ represents the value of input elements after environmental homogenization; max(*Z_i_β_n_*) − *Z_i_β_n_* represents the observable external environmental influencing factors, where Ziβn represents the decision-making unit characteristics used to characterize the worst external environmental conditions, and is the minimum value of the environmental adjustment of each decision-making unit in the whole region, and then it is taken as the evaluation standard, the city area units with better environmental conditions should increase the investment more, and the city area units with relatively poor environmental conditions should increase the investment less, so as to ensure that each decision-making unit is adjusted to the same external environment level; max(*V_ni_*) − *V_ni_* is used to adjust the random error factor, forcing each decision-making unit to be subject to the same external influence.

#### 3.2.2. Industrial Transformation Measurement Model

Referring to conceptual and calculation of the industrial transformation proposed by Findeisen and Südekum [36], we measure the multi-phase industrial transformation range of the Yangtze River economic belt based on the inter-industry reallocation intensity of employees in the research year and the base year. The basic formula is expressed as follows:

(4) $$ISR=\left\{[\sum_{i=1}^{n}\left|L(i,\;t+1)-L(i,\;t)\right|]-\left|L(t+1)-L(t)\right|\right\}/L(t)$$

where, *L* (*i, t* + 1) and *L(i, t)* are used to represent the number of employed persons in the *i*th industry of a certain urban unit in period t+1 and period t respectively; *L*(*t* + 1) and *L*(*t*) are used to represent the total number of employed persons in the urban unit during *t* + 1 and *t* periods.

The Lilien index, which is used to characterize the speed of industrial transformation and upgrading, and the industrial structure adjustment range, which is used to characterize the extent of industrial transformation and upgrading, have certain similarities in terms of scientific connotation. Both of them quantitatively measure the characteristics of industrial transformation and upgrading based on the idea of “the alienation of production efficiency under industrial transformation and upgrading has caused the industrial transfer of labor force”. The former pays more attention to the differences in the labor force of different industries and the whole industry; the latter highlights the inter-annual change of the total labor force of different industries, and more intuitively presents the time characteristics of the speed of industrial transformation and upgrading. In this study, we used the Lilien index and the natural breakpoint method to show the temporal evolution and spatial heterogeneity of the industrial transformation speed. The specific calculation formula of the Lilien index is as follows [37]:

(5) $$L_{t}=\left|{\left[\sum_{i=1}^{n}\frac{L(i,\;t)}{L(t)}{\left(\Delta \log L(i,\;t)-\Delta \log L(t)\right)}^{2}\right]}^{1/2}\right|$$

where, the Lilien index, which represents the speed of industrial structure, is a non-negative value, and the faster the value of this indicator in a specific time period, the faster the industrial redistribution of labor.

The change intensity in the industrial structure is expressed by means of spatial vector analysis based on the changing characteristics of the industrial output value. Specifically, since the *i*-dimensional vector represents the industry during the construction period, the angle between the vectors is estimated to represent the change intensity in the industrial structure, which can intuitively reflect the deep characteristics of industrial transformation and upgrading. The Moore index (α angle) measurement formula based on this idea is expressed as follows [38]:

(6) $$\alpha_{t}=\arccos[\sum_{i=1}^{n}\left(GDP_{i0}\times GDP_{it}\right)/{\left(\sum_{i=1}^{n}GDP_{i0}{}^{2}\times \sum_{i=1}^{n}GDP_{it}{}^{2}\right)}^{1/2}]$$

In the formula, *GDP_i_*_0_ and *GDP_it_* are used to represent the proportion of industrial output value of a city unit in the base period and *t* period, respectively. The larger the value of the Moore index, the faster the industrial output structure changes and the more profound the structural changes. And then the natural breakpoint method is also used as an important means of geographic space-time visualization to intuitively reflect the changes in the proportion of industrial output value between the base year and the target year.

#### 3.2.3. Panel Quantile Regression Model

The quantile regression model proposed by Koenker and Bassett [39] effectively overcomes the above shortcomings. This model is a linear regression model based on conditional distribution fitting, which can identify the non-uniform characteristics of explained variables at different levels and intervals, and accordingly has a certain ability to estimate parameter estimators robustly. The basic model is set as follows:

(7) $$Q_{\tau }(y_{i,\;t}\left|x_{i,\;t},\;control_{t}\right.)=\beta_{\tau ,\;0}+\beta_{\tau }x_{i,\;t}+\gamma_{\tau }^{t}control_{t}+\varepsilon_{t}$$

In the formula, *y_i,t_* represents the explained variable to be used to analyze other external factors; τ represents the quantile value set under different regressions; *x_i,t_* represents the core factor used to analyze the correlation characteristics of the explained variable, that is, the external driving force for the change of the explained variable; *control_t_* means a control variable that has a deep influence on the explained variable and needs to be introduced into an equation to avoid the risk of bias in the potential estimation result of the element, which needs to be combined with scientific issues and objective reality to ensure accurate estimation of parameters; *ε_t_* represents the random error term inherent in the model; and *β* and *γ* represent the coefficients of the corresponding variables, respectively.

#### 3.2.4. Geographically Weighted Regression Model

The geographically weighted regression model attempts to construct a geographic-spatial relationship capture model of the dependent and independent variables with non-stationary characteristics of the parameter to be estimated [40] based on Tobler’s first law of geography [41]. The model structure is expressed as follows:

(8) $$y_{t}=\beta_{0}(u_{i},\;v_{i})+\sum_{m=1}^{p}\beta_{m}(u_{i},\;v_{i})x_{i,\;m}+\sum_{n=1}^{q}\beta_{m}(u_{i},\;v_{i})\cdot control_{i,\;m}+\varepsilon_{i}$$

where, *y_i_*, *x_i_*, control, and *ε* have the same meaning as the corresponding parameters expressed by the quantile regression model formula, that is, they represent the explained variables to be analyzed, the explanatory variables used to explain scientific problems, the control variables used to avoid estimation bias and the random error terms inherent in the model; (*u_i_*, *v_i_*) represents the geographic coordinates of the sample unit *i* under the analysis framework; *β_0_*(*u_i_*, *v_i_*) represents the equation intercept term in the geographically weighted regression model; *β*(*u_i_*, *v_i_*) represents the specific value of the continuous function *β*(*u*, *v*) that falls in the sample unit *i*. Since the model admits that the existing elements have spatial differentiation characteristics, it is necessary to adopt the AIC information criterion as a model selection method, and the optimal criterion is that its value is less than the least squares value by more than 3 [42].

## 4. Results

### 4.1. The Spatiotemporal Differentiation of Urban Economic Efficiency

**Figure 2:**
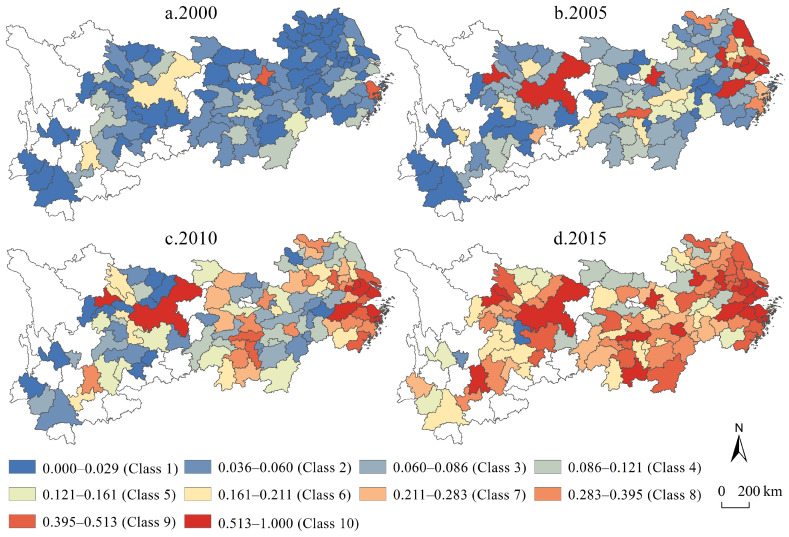
The urban economic efficiency in the Yangtze River economic belt.

In the second stage of analysis, the likelihood ratio test (LR test) of the SFA model of each input element was 387.75, 146.04 and 302.78 respectively, all of which passed the chi-square test with significance at the 1% level, which indicated that the research results of the entire model were basically credible and can be used for analysis. Based on the visual expression of urban economic efficiency in multiple periods (Figure 2), from the perspective of time dimension, the average efficiencies of the four periods from 2000 to 2015 were 0.05, 0.15, 0.20 and 0.33, respectively, and the efficiency values increased by 189.33%, 38.06% and 65.82% in each time period, respectively, showing a non-linear high-speed growth trend overall, which is different from the conclusion that the efficiency value maintains a steady growth or decline in various efficiency analyses based on the SFA model, and fully reveals the fluctuation and rising characteristics in the process of urban economic efficiency improvement. From the perspective of the time dimension, in 2000, the value of urban economic efficiency was low in the whole region, and the spatial homogeneity of efficiency was extremely explicit, and only some cities with relatively high efficiency existed in mosaic form; in 2015, the urban economic efficiency showed a relatively high value in the whole region, forming a high-value city cluster with Shanghai, Wuhan and Chengdu-Chongqing as the core and was W-shaped in form, and showing the center-periphery characteristics that are inverse to the change of geographical distance. In general, the succession of urban economic efficiency in the Yangtze River economic belt has the characteristics of low absolute value, rapid growth period by period, and obvious inter-regional differences.

### 4.2. The Spatiotemporal Differentiation of Industrial Transformation

#### 4.2.1. The Range Pattern of Industrial Transformation

**Figure 3:**
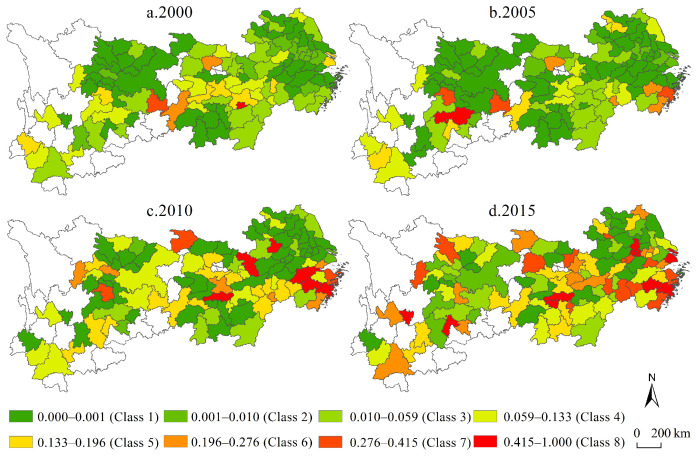
The range pattern of industrial transformation in the Yangtze River economic belt.

From the perspective of the time dimension (Figure 3), the average rate of the industrial transformation range has increased from 0.04 in 2000 to 0.16 in 2015, and the average annual growth rate is as high as 9.13%, which indicates that the rate of industrial transformation and upgrading has increased over a long time span. From the perspective of the spatial pattern, the upstream, midstream and downstream of the industrial transformation range of the Yangtze River economic belt has achieved relatively high growth rates, but there is no obvious spatial agglomeration feature. The specific performance is that the high-value units have gradually changed from the mosaic structure in 2000 to the uniform distribution structure in 2015, indicating that the direct linear correlation between the economic development level of urban units and the reconfiguration intensity of employees is small, and the cities with extremely high and extremely low economic development levels are mostly presented as units with low values in the industrial transformation range.

#### 4.2.2. The Speed Pattern of Industrial Transformation 

**Figure 4:**
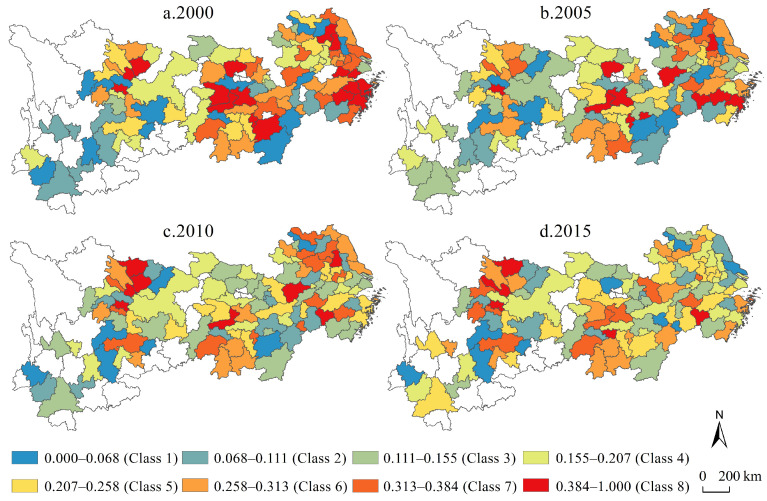
The speed pattern of industrial transformation in the Yangtze River economic belt.

From the perspective of time (Figure 4), the average industrial transformation speed gradually decreased from 0.24 in 2000 to 0.21 in 2015, with a cumulative decrease of 15.77% during the study period. Among them, the class 7 and class 8 (high-value units) decreased from 31 in 2000 to 17 in 2015, while the class 1 and class 2 (low-value units) decreased from 28 in 2000 to 20 in 2015. The simultaneous reduction of high-value units and low-value units means that the industrial transformation speed in the Yangtze River economic belt is gradually converging to the intermediate level, and there is a characteristic of neutral convergence, while the extremely high value and extremely low value units are both unstable forms of industrial transformation over a long-time span. From the perspective of spatial differentiation, the industrial transformation speed of the upper reaches of the Yangtze River and the middle and lower reaches of the Yangtze River show trend deviation and numerical convergence. At the same time, the industrial transformation speed has formed a spatial distribution pattern of low-value agglomeration in the upper reaches of the Yangtze River and high-value agglomeration in the middle and lower reaches. However, the observation of supplementing the time dimension in the spatial perspective shows that the high- and low-value agglomeration characteristics of the industrial transformation speed gradually weaken, while the homogeneous characteristics of the spatial random mixing of high-value cells and low-value cells are enhanced.

#### 4.2.3. The Intensity Pattern of Industrial Transformation

**Figure 5:**
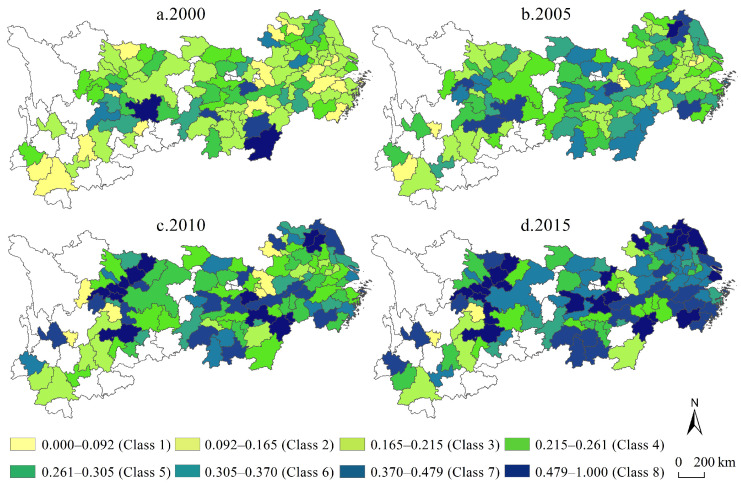
The intensity pattern of industrial transformation in the Yangtze River economic belt.

From the perspective of time (Figure 5), the average value of industrial transformation intensity in the four periods from 2000 to 2015 was 0.17, 0.22, 0.29 and 0.34, respectively, with a cumulative increase of 100.96% over the entire study period. The class 7 and class 8 of industrial transformation intensity increased from 5 in 2000 to 43 in 2015, while class 1 and class 2 decreased from 65 in 2000 to 13 in 2015, indicating that the low tail value was further narrowed and that the head height value increased rapidly. From the perspective of spatial differentiation characteristics, the high-value units in 2000 were mostly concentrated along the Yangtze River, forming a spatial “band-like” connection feature; at the same time, the high-value units of industrial structure change in 2015 have formed a relatively large-scale area structure, forming three peaks centered on the Yangtze River Delta, Wuhan urban circle-Changsha-Zhuzhou-Xiangtan urban agglomeration and Chengdu-Chongqing urban agglomeration, while the western part of Hunan and the central and southern parts of Yunnan have become low-value islands. The results show that based on the industrial transformation intensity, the middle reaches of the Yangtze River economic belt show a relatively rapid cumulative growth disadvantage in a long-term series, which is basically consistent with the subjective perception of social development characteristics of the central collapse in the long-term. In general, the industrial transformation intensity in the Yangtze River economic belt is characterized by spatial agglomeration and temporal divergence.

### 4.3. The Driving Mechanism of Industrial Transformation on Urban Economic Efficiency

#### 4.3.1. Piecewise Analysis of Panel Quantile Regression

Based on the parameter estimation of the panel quantile regression model (Table 1), it can be seen that the parameter estimates of industrial transformation range (*β*_1_) and industrial transformation speed (*β*_2_) showed that the obviously characteristics of the parameters increased from strong in the low quantile to very strong in the middle quantile and then declined to weakly in the high quantile; the parameter estimates of industrial structure intensity (*β*_3_) and intellectual capital level (*γ*_1_) have similar trends, and they can still maintain a high level of significance at high quantiles; the parameter estimates of the government intervention degree (*γ*_2_) and the urban-rural income gap (*γ*_3_) increased from very indistinct fluctuations in the low quantiles to more explicit ones in the high quantiles. The increasing or inverted U-shaped evolution law of the significance level of the parameters to be estimated above reveals that the analysis of the evolution mechanism of urban economic efficiency between divisions, tiers and stages has higher practical guiding significance.

**Figure 6:**
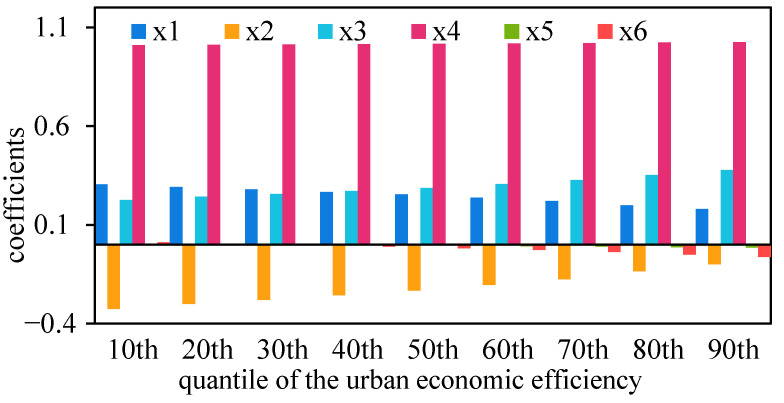
Variation diagram of the panel quantile regression coefficient.

On the basis of the table display of the parameter estimation results of the panel quantile regression model, the quantile change graph of the estimated value of each parameter is drawn to form an intuitive expression of the evolution characteristics of the parameter coefficient (Figure 6). From the perspective of specific parameters, the index coefficient value of the industrial transformation range gradually decreased, from 0.31 in the 10% to 0.18 in the 90%, with a cumulative reduction of 41.05% in the positive promotion effect; the industrial transformation speed index increased from −0.32 in the 10% to −0.10 in the 90%, and the negative inhibitory effect decreased by 69.70% cumulatively; the index of industrial structure intensity increased from 0.23 in the 10% to 0.38 in the 90%, with a cumulative growth of 66.98%; the intellectual capital level index increased from 1.01 in the 10% to 1.03 in the 90%, with a cumulative increase of 1.53% in positive promotion; the index of government intervention degree decreased from 0.0002 in the 10% to −0.02 in the 90%, and the positive effect turned to negative inhibition; the income gap between urban and rural residents decreased from 0.01 in the 10% to −0.06 in the 90%, which also turned from a positive promotion to a negative inhibition.

The characteristics of parameter changes under each quantile are different. For example, the intellectual capital level is high and stable in the full quantile, while government intervention degree and the urban-rural income gap are both small in absolute value, rapid relative change and changing direction of action. It is particularly worth noting that industrial transformation range, industrial transformation speed, and industrial transformation intensity, which are characterized by changes in industrial employment, show the characteristics of alienation and succession. Although industrial transformation range and industrial transformation speed have opposite effects, both of them show a “near zero” change with a decrease in absolute value, while the degree of industrial structure change shows a “far zero” change with an increase in absolute value in the progressive process from low quantiles to high quantiles.

#### 4.3.2. Space-Time Dynamic Mechanism Analysis of Geographically Weighted Regression

The period-by-period values of the AIC information criterion analyzed by the geographically weighted regression model are −315.24, −127.83, −126.54 and −133.23, respectively, while the period-by-period values of the AIC information criterion analyzed by the OLS model are −317.91, −123.07, −123.19 and −124.53, which indicates that the effect of the industrial transformation based on the geographically weighted regression model on the urban economic efficiency has passed the three tests in 2005, 2010 and 2015, and the geographically weighted regression model should be adopted to comprehensively consider the local effects of geographical things to carry out an in-depth analysis of the driving factors. Although the comparison results between models in 2000 show that the spatial differentiation of driving factors is relatively weak, they can be used to compare the evolution characteristics of the estimated values of the multi-period industrial transformation series index coefficients.

**Figure 7:**
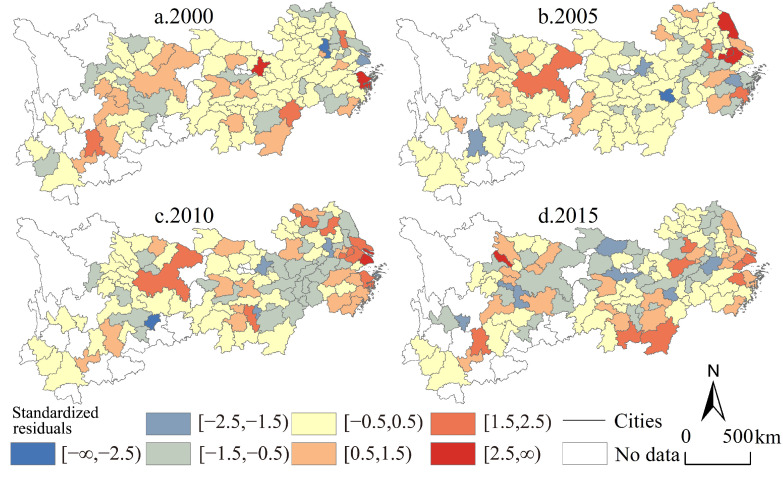
Standardized residuals of the GWR model in the Yangtze River economic belt from 2000 to 2015.

As the basis for judging the estimation effects of parameters in different geographical units, the standardized residual values calculated by the geographically weighted regression model can intuitively reflect the estimation effects of the models at the level of city units and the whole region of the Yangtze River economic belt (Figure 7). From 2000 to 2015, the total number of urban units whose absolute values of standardized residuals were less than 2.5 were 108, 107, 109, and 110, respectively, accounting for 97.30%, 96.40%, 98.20%, and 99.10% of the total urban units, of which the number of city-area units whose absolute value is less than 0.5 are 69, 68, 50, and 43, respectively, accounting for 62.16%, 61.26%, 45.05%, and 38.74% of the total city-area units, respectively. The above results all show the applicability and scientificity of using the geographically weighted regression model to carry out industrial transformation on the analysis of the urban economic efficiency improvement mechanism, especially in the analysis of the estimated value of each parameter and its effect degree, which has high explanatory power and credibility.

**Figure 8:**
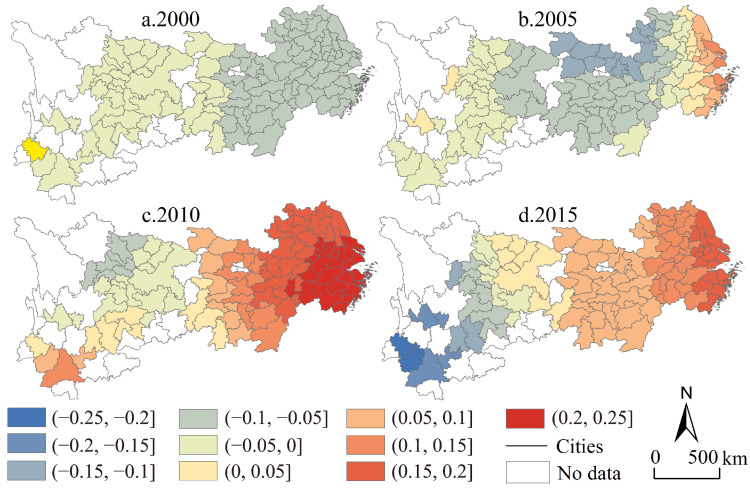
Regional coefficient map of industrial transformation range in the Yangtze River economic belt from 2000 to 2015.

On the basis of the double test of the model’s applicability and explanatory power, based on the statistical distribution characteristics of the estimated industrial transformation range and considering the direction of their driving effects, a classification map of parameter estimates in equal intervals was formed with a value of 0 as the critical point (Figure 8). From the perspective of sub-category characteristics, the characteristics of regional differences in the extent of the industrial transformation range have gradually increased. In 2000, there were only two negative clusters in the whole region, and the estimated coefficients of the transformation and upgrading of the unit industries in each city area tended to be the same; in 2015, the scale of industrial transformation range in the whole region increased rapidly, and the whole region could be divided into the relatively obvious spatial clustering of eight-level units, and showed a relatively obvious numerical decreasing law from coastal to inland. Combined with the fact that the geographically weighted regression model in 2000 failed to pass the model hypothesis test, it shows that from 2000 to 2015, the spatial non-stationary characteristics of the extent of industrial transformation range increased rapidly, thus forming an urban economic efficiency mechanism with enhanced local effects. From the perspective of time and space, in 2005, the industrial transformation range showed a U-shaped pattern with a low value in the middle, followed by the west, and a higher value in the east; in 2010, it formed with Shanghai, Zhejiang and Jiangsu as the core, showing the law of decreasing positive effect capacity from the core to the periphery, and finally appeared to have a negative value in the western region; the characteristics of spatial differentiation in 2015 were similar to those in 2010, and the units with absolute high values of industrial transformation range coefficients showed an inverted U-shaped development law of growth and weakening. In general, the local negative-acting units show a numerical transition from strong negative to weak negative to weak positive, indicating that there are large differences in spatial development among regional unit groups.

**Figure 9:**
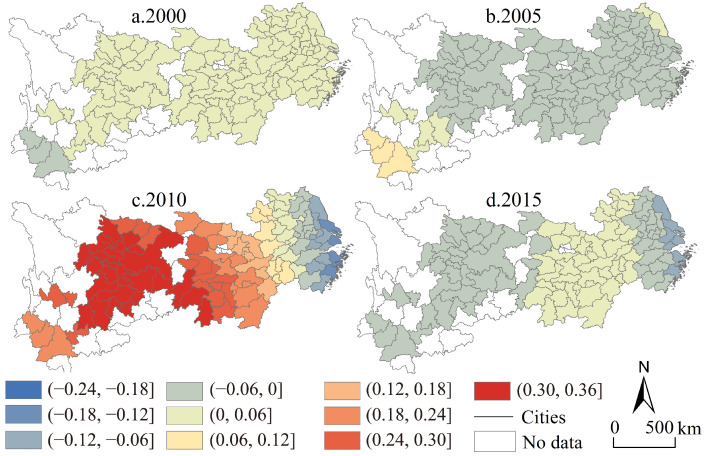
Regional coefficient map of industrial transformation speed in the Yangtze River economic belt from 2000 to 2015.

Using the same mapping method as the coefficient map of the industrial transformation range, a map of the spatiotemporal evolution of the industrial transformation speed in the Yangtze River economic belt from 2000 to 2015 was created (Figure 9). From the perspective of sub-category characteristics, the regional differences of industrial transformation speed also show an increasing trend. In 2000, there was only one type of industrial transformation speed type as the main form in the whole region; in 2015, the classification of the industrial transformation speed in the whole region increased rapidly, and the whole region can be divided into relatively obvious three-level and four-region agglomeration characteristics. From the perspective of sub-regional characteristics, in 2005, the industrial transformation speed was dominated by the absolute negative value of the coefficient, and a few units with positive values in the east and west were symmetrical; in 2010, the central and western regions were the high-value units, gradually decreasing to the eastern region and returning to a negative value; in 2015, it returned to the distribution characteristics of low absolute values of coefficients at both ends and high in the middle. The above differences between years and regions highlight the necessity of space exploration.

**Figure 10:**
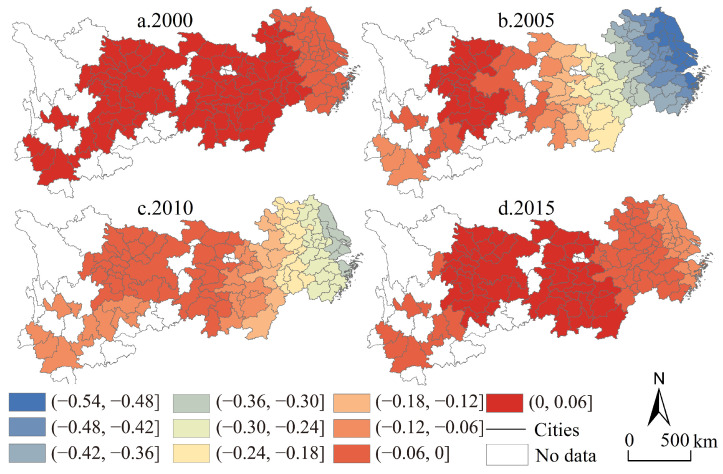
Regional coefficient map of industrial transformation intensity in the Yangtze River economic belt from 2000 to 2015.

Compared with the parameter estimation results of the quantile regression model, it can be seen that there are more positive urban units and more negative urban units at the same time as the degree of change in the observed industrial structure (Figure 10), which is a regional law that cannot be summarized and revealed by the above model. From the perspective of category characteristics, in 2000, there were only two urban clusters in the whole region, and the coefficients of industrial structure change of the two clusters were similar; from 2005 to 2015, the number of categories of coefficients of the degree of change in the global industrial structure was greater, but it showed a trend of decreasing categories. From the perspective of variation characteristics, the characteristics of industrial transformation intensity changes from 2005 to 2015 generally showed low values in the east with Jiangsu Province as the core, middle values with Hubei Province as the core, and the western high value with Sichuan Province as the core, showing a negative effect decreasing from the coast to the inland. From the perspective of development trends, even though there are big differences in the direction and degree of action in the upper, middle and lower reaches of the Yangtze River economic belt, the intensity of industrial transformation shows a trend of period-by-period growth, showing a strong negative-weak-negative, weak-negative-positive succession law of extremely low-value units in space, while spatially-positive units show the U-shaped change characteristic of an initial decrease followed by an increase.

## 5. Discussion

Urban economic efficiency and industrial transformation have always been the key issues of concern in terms of economic geography, regional economics, and land management. It has constantly spawned multi-scale, wide field, and wide region academic rational research, and formed a comprehensive conclusion including time, region, and perspective differences. Through a brief review of these studies, we found that there are two important rules that apply to them. One is that there is still controversy about the changing law of urban economic efficiency. Unlike this study, some studies recognize that urban economic efficiency has a downward trend [43], which is mainly because land resources are not used as an important input factor for urban development. In fact, the research on urban economic efficiency that supplements land resources as a factor input generally recognizes the general upward trend of economic efficiency [44], which indicates that considering the role of land resources in urban economic development may be an important supplement to the innovative understanding of urban development. The second is that the effect of industrial transformation on urban economic efficiency is basically the same [45,46]. The existing research basically recognizes that industrial transformation is beneficial to the improvement of urban economic efficiency, which is consistent with the comprehensive positive effect of industrial transformation in this study. However, the existing research generally adopts a single indicator to describe the industrial transformation, which makes it difficult to reveal the differential effect of the multi-dimensional characteristics of the industrial transformation on the urban economic efficiency, which is also one of the core contributions of this research.

The reanalysis of the driving effect of industrial transformation on urbanization efficiency can be used as a reference for regional planning and linkage strategy formulation of all parties in the Yangtze River economic belt in order to reduce the potential irrational decision-making risk in the description of individual and time characteristics of cities in the Yangtze River economic belt. The main suggestions are as follows: firstly, the urbanization efficiency value is still generally low. It is necessary to combine the characteristics of the new economic normal and regional development conditions, adopt the advantage cultivation of core competitive units, and formulate and implement strong measures to improve urbanization efficiency. Secondly, to strengthen the core leadership of Jiangsu, Zhejiang, and Shanghai, boost the rise of central China, and tap the great potential of Western China, and form a gradient development strategy focusing on quality development in the upper reaches of the Yangtze River economic belt, undertaking upstream and independent creation in the middle reaches, and undertaking industrial transfer in the lower reaches. Finally, it is necessary to strengthen the head effect of industrial transformation range with Shanghai as the core in the upper reaches of the Yangtze River economic belt, the supporting role of industrial transformation speed with Wuhan as the core in the middle reaches, and the strategic guarantee of industrial transformation intensity with Chengdu and Chongqing as the core in the lower reaches.

In addition, the discipline of geography focuses on exploring the systematic laws of nature, economy and society based on the regional, comprehensive, and intersecting attributes of things, which determines that geography in the new era will further focus on complex spatial patterns, complex time processes and complex dynamic mechanisms [47,48]. Among them, because there are a multiplicity of factors, subjective arbitrariness, and irrational decision-making characteristics in the exploration of the laws of human affairs, it is inevitable to form realistic, scientific, and effective judgments and strategic decisions based on a single research result or a single dimension of practical exploration. However, even if the optimal policy of static nodes in urban development always has the risk of deviation from the optimal decision-making of all subjects, the value orientation and goal guidance of comprehensive multiple studies can explore and summarize feasible sub-optimal development strategies in combination with regional reality. However, due to the bias of subjective understanding and the limitation of research boundaries, it is difficult to fully explain the mechanism of analysis of industrial transformation on urban economic efficiency. The following topics may be the focus of future related research: Firstly, since the geographical scale effect widely exists in various geographical things and phenomena, the analysis of the urban economic efficiency improvement or inhibition effect of industrial transformation at different scales and the analysis of the geographical space scale effect can effectively serve the macro policy implications of sub-scale and sub-regional effects, which is also a useful supplement to the condensed interaction effect of geographic scale and transition law. Secondly, the temporal and spatial differentiation characteristics of geoscience phenomena and geographical things change extremely drastically under the double observation of time scale and space scale. If the time scale is gradually reduced to days, hours, and minutes, and the spatial scale is gradually refined to districts, counties, streets, and communities, the risk factors of industrial transformation and urbanization’s macro-representation deviation will be eliminated, and the refined planning and management of spatial decision-making units will be promoted. Finally, based on the hierarchical judgment and node identification of multiple decision-making, qualitative and quantitative methods are used to evaluate the direct effect of industrial policy on industrial transformation and the indirect improvement of urban economic efficiency under strategic needs and decision-making preferences, as well as the potential interactive feedback between the three, and then to reveal the hierarchical and spatial non-stationary characteristics of macro-regulation and micro-governance.

## 6. Conclusions

Based on the scientific perspective of geographic spatial heterogeneity and temporal succession, we take 111 urban units in the Yangtze River economic belt as the research object, construct a city-level database for the fourth phase of urban development and industrial transformation from 2000 to 2015, use three-stage DEA, industrial transformation measurement, panel quantile regression, and geographically weighted regression to analyze the effect of industrial transformation in the Yangtze River economic belt on urban economic efficiency, to expound the spatiotemporal differentiation law of the industrial transformation on the urban economic efficiency, and to reveal its phase imbalance and spatiotemporal non-stationarity characteristics, which finally formed the cognition of the geographical linkage between high-quality urban development and industrial transformation. The main conclusions are as follows,

(1) Based on the numerical calculation and spatiotemporal expression of urban economic efficiency in multiple periods, it is determined that the mean value of city level economic efficiency in the Yangtze River economic belt presents a non-linear high-speed growth trend. At the same time, the urban economic efficiency of the Yangtze River economic belt from 2000 to 2015 has gradually changed from a spatial mosaic to a gradual spatial correlation between the center and the periphery, and based on the multi-year urban economic efficiency comparison, we have concluded that Shanghai, Wuhan, and Chengdu-Chongqing are the cores and are characterized by a w-shaped cluster of local advantageous cities.
(2) From the perspective of industrial transformation characteristics, we analyzed the characteristics of heterogeneous changes in industrial transformation, where industrial transformation range and intensity continued to increase, and industrial transformation speed gradually decreased, and then delineated the area structure in which the industrial transformation range tends to be homogeneous in spatial distribution, the industrial transformation speed tends to converge in the middle, and the industrial transformation intensity tends to spread to regional peaks.
(3) In general, the industrial transformation range and the industrial transformation range intensity have a positive effect on urban economic efficiency, while the industrial transformation speed has a negative effect on urban economic efficiency. With the increase of quantiles, the positive effect of the industrial transformation range and negative effect of the industrial transformation speed are gradually weakened, showing the characteristics of “near zero” change, while the positive effect of the industrial transformation intensity are gradually strengthened, showing the characteristics of “far zero” change.
(4) We conclude the spatial local effects of the factors affecting urban economic efficiency and the explicitly spatial non-stationary characteristics of the driving mechanism, and then summarize the time-inverted U-shaped evolution of the industrial transformation range, the inverted U-shaped pattern in time and space of the industrial transformation speed, and the time-continuous growth and the spatially inverted U-shaped distribution characteristics of the industrial transformation intensity.

## Figures and Tables

**Table 1:** Parameter estimation results of the panel quantile regression model.

Parameter	10th	20th	30th	40th	50th	60th	70th	80th	90th
*β*_1_	0.305 ***	0.291 ***	0.280 ***	0.267 ***	0.254 ***	0.238 ***	0.221 ***	0.199 **	0.180
	(2.95)	(3.33)	(3.68)	(4.09)	(4.29)	(3.99)	(3.20)	(2.19)	(1.56)
*β*_2_	−0.324 **	−0.300 ***	−0.280 ***	−0.256 ***	−0.233 ***	−0.204 ***	−0.174 **	−0.134	−0.098
	(−2.49)	(−2.72)	(−2.91)	(−3.10)	(−3.11)	(−2.70)	(−1.99)	(−1.16)	(−0.68)
*β*_3_	0.226 **	0.242 ***	0.256 ***	0.272 ***	0.287 ***	0.306 ***	0.326 ***	0.353 ***	0.377 ***
	(2.11)	(2.68)	(3.24)	(4.01)	(4.68)	(4.95)	(4.55)	(3.74)	(3.17)
*γ*_4_	1.010 ***	1.011 ***	1.013 ***	1.015 ***	1.016 ***	1.018 ***	1.020 ***	1.023 ***	1.025 ***
	(3.16)	(3.74)	(4.31)	(5.04)	(5.57)	(5.53)	(4.78)	(3.64)	(2.89)
*γ*_5_	0.0002	−0.002	−0.003	−0.005	−0.006	−0.008 **	−0.010 **	−0.013 **	−0.016 **
	(0.03)	(−0.25)	(−0.55)	(−1.01)	(−1.51)	(−1.98)	(−2.15)	(−2.08)	(−1.97)
*γ*_6_	0.011	0.004	−0.003	−0.011	−0.019	−0.028 **	−0.039 **	−0.051 **	−0.063 **
	(0.48)	(0.17)	(−0.17)	(−0.71)	(−1.33)	(−1.99)	(−2.32)	(−2.39)	(−2.34)
*N*	444	444	444	444	444	444	444	444	444

** *p* < 0.05, *** *p* < 0.01.

## Data Availability

Not applicable.
